# Time Spent in Practicing Dissection Correlated with Improvement in Anatomical Knowledge of Students: Experimental Study in an Integrated Learning Program

**DOI:** 10.7759/cureus.7558

**Published:** 2020-04-06

**Authors:** Hussein Abdellatif

**Affiliations:** 1 Human and Clinical Anatomy, Sultan Qaboos University, College of Medicine and Health Sciences, Muscat, OMN; 2 Anatomy and Embryology, Faculty of Medicine, University of Mansoura, Mansoura, EGY

**Keywords:** assessment results, cadavers, dissection, learning, students

## Abstract

Introduction and aim

Cadaveric dissection has long been used as the main domain for teaching anatomy in medical schools. However, recently with a limited number of cadavers and time for practicing dissection, prosections and anatomical models are widely used and may replace traditional dissection. We aimed to explore the possible association between practicing dissection and test results among medical students and to determine whether there are differences in achievements between students who studied anatomy by cadaveric dissection and those who used prosections and anatomical models.

Methods

The study was conducted at the University of Bisha, College of Medicine, Saudi Arabia, during the period from March to August 2017. Students were randomly assigned to one of two groups (50 in each). The first group studied anatomy (upper limb course) by practicing dissection while the other studied it by using prosections and anatomical models. Both groups were subjected to the same final assessments. Scores of both groups were compared by using the Student’s t-test. Correlation analysis between time spent in practicing dissection (carefully registered using a predesigned portfolio and an attendance logbook) and assessment grades was implemented using the rank-based Pearson correlation coefficient.

Results

Students practicing dissection achieved higher grades (169 ± 1.99) than those who studied anatomy by only using prosections and anatomical models (142 ± 1.78, p<0.001). There was an association between the time spent in practicing dissections and overall anatomy summative assessments (r^2^=0.841, p<0.001). Students expressed positive responses towards the effectiveness and value of practicing dissection.

Conclusions

We concluded that practicing dissection helps students to achieve higher results than learning using only models and prosections. Time spent in practicing dissection correlated with final assessment results. Further research is required to measure not only the statistical significance of results but also their educational effectiveness and long term learning outcomes.

## Introduction

Cadaveric dissection has been the principal domain of anatomy teaching since the 16th and 17th centuries [1]. Anatomical knowledge is fundamental for developing the clinical skills of students, particularly in practicing surgery. The teaching process needs continuous revision and updates to determine approaches that best suit the changes in curriculum and learning processes. Dissection was considered the most accepted method for professional training and skill development in future doctors [2]. The process of dissection provides students with an important three-dimensional view of the human body, which not only enhances regional and system anatomy knowledge but further reinforces their knowledge acquired from lectures and tutorials [3]. The study of the human subject may provide an opportunity to understand the range of variability present in real human material as compared to that described in textbooks and plastic specimens. Working in groups in the dissecting hall would not only foster teamwork, encourage peer and self-directed learning but would also inculcate professionalism and respect for the cadaver as a valuable learning resource [4].

Owing to debates on balancing learning outcomes, difficulties related to the use of human cadaver, teaching methods, and resources, many recent curricula in anatomy have suggested a shift towards intense use of alternative teaching modalities involving cadaveric plastination, prosections, non-cadaveric models and computer-based imaging [5].

Of these new teaching modalities, demonstration using models and prosections are currently the most popular and applicable methods for teaching anatomy in medical schools. A prosection is a previously dissected cadaveric material. Cadaver prosections were an essential method of teaching anatomy in the Middle Ages and early Renaissance [6]. With a limited number of donated bodies and a decrease of time allocated for practicing dissection in modern integrated curricula, many programs have moved from full-body dissection to using prosections. This reduces the number of contact hours and allows learners to explore more the structures that they might otherwise spend hours trying to find and explore in dissection classes [7, 8].

Furthermore, in deeply located organs as the heart, dissection for such organs may be quite difficult and time-consuming. Furthermore, in a system based approach to teaching anatomy, the role of dissection, which is essentially regional, loses significance. Hence using prosections and fiberglass models for demonstrations would prove useful. Many surveys recently conducted in medical schools using integrated, systemic, and case-based learning showed that prosections are commonly used for teaching anatomy rather than whole cadaveric dissection [9]. However, many anatomists still favor the use of dissection over other teaching tools. A study by Kerby et al. stated that dissection is the “fit for purpose” in meeting the learning outcomes, but no single method met all fields and aspects of the curriculum [9]. Thus, there has been a debate on whether the use of prosections and anatomical models is more effective in learning anatomy and may replace traditional dissection in modern integrated curricula [10]. 

In this article, we aimed to compare assessment outcomes between students who studied anatomy by using cadaveric dissection and those who studied it by only using prosections and anatomical models and to demonstrate if there is a possible association between time spent in practicing dissection or studying prosections and anatomical models and the final anatomy course results of medical students.

## Materials and methods

This randomized experimental study was conducted among medical students (phase I, second year) in the College of Medicine (male-only institution), University of Bisha (UBCOM), Saudi Arabia, during the period from March to August 2017. Informed consent for study participation was implied upon starting the study. The confidentiality of the study participants was maintained throughout the study. The ethical review board at the University of Bisha, College of Medicine approved the study.

The study population included a random sample of phase I medical students, who would start their elective anatomy courses in their second academic year. These courses were optional for students, and their results would not be included in their final academic scores, it had been used as an index of general academic ability (thus it was used as an informative and not a summative form of assessment to direct students and staff about the efficacy of the used curriculum and the reliability and validity of the utilized teaching and assessment methods). Participation in the study was voluntary and was offered as a complementary learning activity during summer. All the participating students had finished anatomy courses in their first academic year according to the teaching curriculum. The educational program in UBCOM is integrated and utilizes a self-directed learning program (SDL), composed of block-based modules that depend on hybrid problem-based learning (PBL), as the primary learning tool. The UBCOM undergraduate program is five years, spread over three phases of learning (basic medical sciences - phase I; pre-clerkship - phase II; and the clerkship - phase III). Anatomy classes are distributed throughout the whole course according to the planned outcomes included in each module. Anatomy in all phases is taught by traditional lectures and practical sessions. Phase I modules with anatomy classes are 1) basic structure and function of the human body; 2) growth, development, and aging. Students in these classes study the anatomical basis of the human body, anatomical terms and planes, and different phases of human life. The students learn about stages of development and embryological growth. Students in this phase did not practice dissection and only used prosections and anatomical models in their practical sessions.

One hundred medical students from the second academic year (phase I) were included in the study. The sample size of 100 participants was calculated using the following formula: ss =Z 2 * (p) * (1-p) / C2. Where ss is the sample size, Z is the Z value (1.96 for 95% confidence level); p is the percentage picking a choice (expressed as a decimal, 0.5), and C is the confidence interval (8.77).

The cohort was randomized into two parallel teaching formats: group 1 (dissection group) and group 2 (prosections and models’ group). The choice of the upper limb for the study was decided due to its place in the learning curriculum and to the necessity of both groups to practice an organized regional dissection in order to get an opportunity for comparison between them at the end of the study. 

Students were assigned for groups by simple random sampling after acquiring the list of students from the college administration. Computer-generated random numbers were used for simple randomization of subjects [11].

The study guide was distributed among students with the same learning objectives between both groups. All the learning outcomes were covered in the practical classes and interactive lectures. The main features of the experimental program were the same for both groups apart from studying the practical sessions of anatomy. The study of the upper limb was divided into 16 headings, including soft tissues, bones, and joints of the upper limb (Table 1).

**Table 1:** Upper limb subject areas

List of topics	
Bones of upper limb	Flexor retinaculum and carpal tunnel
Pectoral region and breast	Superficial muscles of the back of the forearm
Muscles of shoulder and scapular region	Deep muscles of the back of the forearm
Intermuscular spaces in shoulder region	Anatomical snuffbox and extensor retinaculum
Axilla	The hand
Arm and superficial muscles of the front of forearm	Blood vessels of upper limb
Deep muscles of the front of forearm	Nerves of upper limb
Cubital fossa	Joints of upper limb

The headings were taken one per each practical class. Students were advised to use the reference books mentioned in the study guide as a preparatory tool before attending the anatomy sessions. All practical classes started with priming sessions in tutorial rooms lasting for 30 minutes at which the work plan was defined.

Students were then divided into two shifts of 25 each in succession to the dissection room (two cadavers were used - one male and one female) where students practiced dissection in succession. The material was demonstrated to group 2 by an instructor using prosections and anatomical models whereas, in group 1, students started dissection using a dissecting manual and demonstrated exposed structures to one another. Staff attended the class and supervised students by answering the questions and participating in discussions. Students were free to ask for help from staff at any time. Practical sessions were conducted twice per week for eight learning weeks (three hours each). Both groups (dissection and prosections) attended the same lectures and preparatory tutorials; the difference was in the use of dissection or prosections in their practical classes.

Students’ attendance in practical courses was carefully registered using a predesigned portfolio and student’s logbook. The supporting staff in the dissection room recorded all the entries and the dissection practice for each student individually. The attendance was recorded at the end of each session in order to make sure that students have attended the classes from the beginning till the end and did not leave the class after marking their names at entry. Those who left the session due to an excuse or before the end were carefully registered as well. Students in both groups were allowed freely enter to the anatomy laboratory. Students in group 1 had free access to practice dissection outside their teaching time. The time spent in practicing dissection outside teaching hours was carefully registered for each student separately. Students in group 2 could go and visit prosections as often as needed; the time they spent in the anatomy laboratory was registered as well.

On completing the course, both dissection and prosection groups were submitted to two tests: a theory test of 60 multiple-choice questions (MCQs) giving two marks each, and a practical exam of 20 stations (each station had one labeled structure for identification and one additional question related to specimen) giving four marks each. Out of these 60 questions, 45 MCQs were selected from the question bank and 15 questions were constructed before the exam. Questions were selected from the bank based on analysis reports of previous exams. Questions with a discrimination index of 0.3 or more were used and those with lower indices were excluded.

Measures of test reliability, validity, and variance among scores were carefully considered during test construction and selection of items from the test bank. Test-retest reliability, internal consistency (Cronbach’s alpha and KR-20 ≥ 0.7), and inter-rater reliability indices were considered. For measures of test validity, both construct and content related validity were carefully considered in item selection (validity coefficient (r) ≥ 0.3).

Scoring of the final exam (both theory and practical) concluded 200 marks. In the practical exam, 20 structures (one structure in each station) were labeled using pins or robes. In theory exam, 60 multiple choice questions were assigned. The assessment time was the same for both groups. Questions varied in their difficulty from simple questions that measure the knowledge to more complicated ones that measure higher cognitive learning domains. The sample of exam questions and their relation to learning domains are presented in Table 2.

**Table 2:** Multiple-choice questions in relation to learning outcomes and domains

Topic title:	Specific learning outcomes	Learning Domains		
		Knowledge	Cognitive	Psychomotor
Anatomy of axilla	1. List the boundaries of the axilla. Which of the following muscles is present in the anterior wall of axilla?	Multiple choice question		
	A. Teres major			
	B. Latissimus dorsi			
	C. Subscapularis			
	D. Pectoralis major			
	2. Enumerate the contents of the axilla. Which of the following is content in the axilla?	Multiple choice question		
	A. Roots of brachial plexus			
	B. Trunks of brachial plexus			
	C. Cords of brachial plexus			
	D. Branches of cervical plexus			
	3. Explain the formation of the brachial plexus and its branches. A 29-year-old man comes in with a stab wound, cannot raise his arm above horizontal, and exhibits paralysis of long thoracic nerve, Which of the following structures of the brachial plexus would most likely be damaged?		Multiple choice question	
	A. Medial cord			
	B. Posterior cord			
	C. Lower trunk			
	D. Roots			
	4. Demonstrate the origin, course, branches and termination of the axillary artery. Which of the following is A branch of the first part of axillary artery?	Multiple choice question		
	A. lateral thoracic			
	B. Acromiothoracic			
	C. Superior thoracic			
	D. Subscapular			
	5. Identify different groups of axillary lymph nodes and their drainage The apical group of right axillary lymph nodes drains into which of the following?	Multiple choice question		
	A. Thoracic duct			
	B. Right lymphatic duct			
	C. Cisterna chyli			
	D. Right mediastinal lymph nodes			

In the practical exam, we used prosected and readily dissected specimens for each group separately. Both dissected specimens and prosections were prepared to show both the superficial and deep layers of arm, forearm, and hand muscles. Bone identification was on a real skeleton. Examples of labeled structures were supinator, brachioradialis, and flexor digitorum superficialis. Identification of axillary, radial, ulnar and superficial palmar arches were also included. Questions ranged in their complexity from very simple questions assessing only the knowledge like muscle identification to more complicated ones like the effect of the nerve injuries and types of resulting deformities that measure understanding and cognitive domains. Students scored maximum of 80 marks. Students in both groups performed the test individually and were allowed to manipulate the specimens. Questions were the same for both groups, and they performed the test simultaneously in identical stations. The exam was corrected by two independent staff members, and the inter-rater reliability was calculated.

The demographic parameters of students were collected and analyzed. Quantitative variables were presented as the mean and standard error of the mean. P-value < 0.05 was considered significant. Association between variables was assessed by Pearson's correlation coefficient. The effect size was calculated using Cohen’s equation: d = Mean 1 (gp1) - Mean 2 (gp2)/ Avg SD. Where Avg SD is the average of both standard deviations. Cohen’s d of 0 to 0.2 standard deviations means small effect, 0.2 to 0.5 medium effects, and > 0.5 large effects. Statistical analysis of data was carried out with SPSS version 17.0 (SPSS Inc., Chicago, US ). 

## Results

One hundred male students participated in the study. All of them (100%) took the final exam, and no absence was recorded. The mean age of participants was 18 ± 0.76. Participants were divided into two groups. The time allocated for practical sessions was 48 hours for both groups. Time spent by each student in practicing dissection (group 1) or in studying the prosections and models (group 2) was individually calculated.

**Figure 1:**
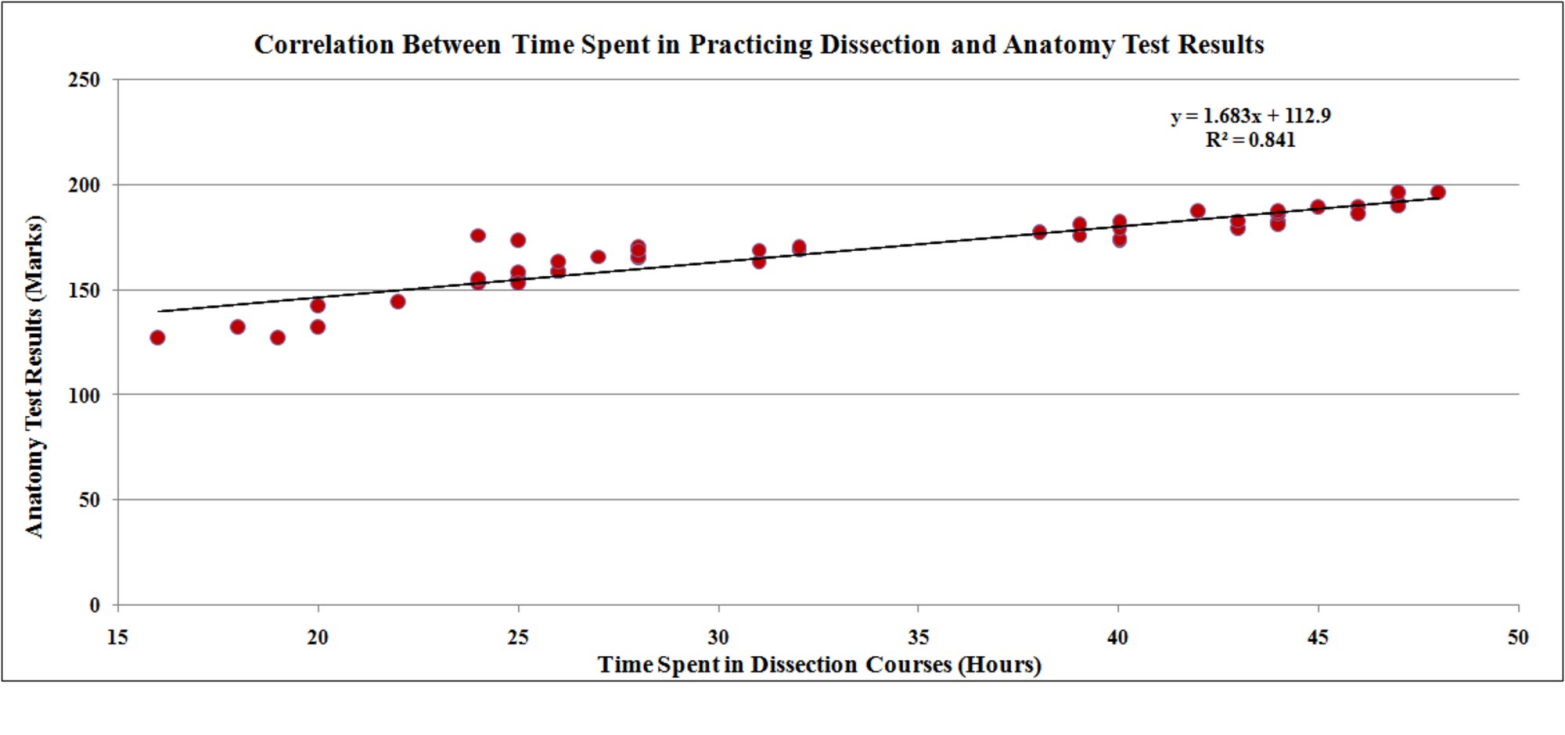
Correlation between assessment results of students in the final exam and the time spent in dissection courses for the dissection group

Group 1 outperformed group 2 in both tests with mean results of 169 ± 1.99 and 142± 1.78, respectively. The difference between groups was significant at the 0.05 level. The dissection group 1 did better in theory and practical tests compared to the prosections and models’ group 2 (Cohen’s d=2.08) with a significant difference between two groups. There was a positive correlation between time spent in dissecting cadavers and the final anatomy course assessment results (r^2 ^=0.841, p <0.001) in the dissection group (Figure 1).

**Figure 2:**
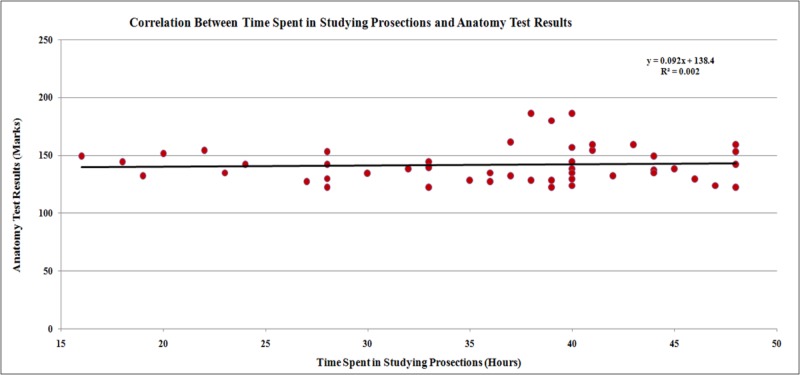
Correlation between assessment results of students in the final exam and the time spent in contact with prosections for the prosections and models' group

However, in the prosections and models' group such correlation between overall times spent in practical sessions and in studying physical materials and the assessment results was not found (r^2^=0.002, p=0.735) (Figure 2).

Test item analysis was performed to ensure the achievement of test validity and reliability (KR-20=0.796). The inter-rater reliability was calculated as two independent members revised the practical exam results, Using the Pearson’s correlation, scores of one member were correlated with scores from the other member for each exam result and was found to be 0.846 (85%) showing that two ratings had a high match. 

## Discussion

It is well known that students learn anatomy from the dead bodies already since the Renaissance [7]. However, a limited number of cadavers and lack of legislation that allowed students to learn anatomy by practicing dissection has remained an obstacle to its routine use in anatomy teaching. After the enforcement of the anatomy act, sufficient cadavers have been available for dissection practice in most medical schools. Demonstration to students by dissecting cadavers and students’ own dissection practice is currently the traditional anatomy teaching method [12]. However, the low availability of resources and reduced time allocated for teaching anatomy in modern curricula have led to a shift from traditional dissection towards modern and innovative methods of teaching. Improvement in preservation techniques, creation of plastinated specimens, and rapidly advancing technology in medical education have increased the use of prosected specimens and multimedia-based methods in teaching anatomy [13, 14]. 

Though dissection remains a mainstay in the process of anatomy teaching, several institutions have advocated its removal from modern teaching medical and allied health curricula for the following reasons: reduced allocated time for dissection, limited availability of resources, lack of possible donors, unpleasant smell of cadavers and shift towards the use of modern technologies in teaching [9, 15-19]. This led to a debate among anatomists regarding the optimal method for delivering anatomy to medical students and their desired degree of anatomical competence.

Literature reviews reveal that there are varying perceptions towards attitudes, emotions, and the necessities of medical students to learn through cadaveric dissection. More research in anatomical educational methods is necessary to arrive at a consensus about the optimum methodologies regarding the imparting of anatomy education, based on scientific evidence. Winkelmann et al. noted that while planning dissection courses as a learning tool in anatomy, one needs to consider that it is not a uniform learning experience, and participants within the same course may seek divergent learning tools [20]. This goes in line with Wilson and his colleagues who performed a meta-analysis on anatomy pedagogies in laboratories, and they concluded that there is still a debate about the advantages and disadvantages of using different modalities in anatomy teaching. They found no significant difference in students’ performance when comparing traditional dissection with other laboratory methods (prosections, digital-media, and hybrid approaches). The difference in results between our work and their results may be attributed to differences in learning curricula (PBL based curriculum versus traditional lecture-based learning); besides our study was conducted in the summertime where students were freer to practice dissection or study using prosections [21]. 

Lack of standardization between various medical courses and teaching curricula makes it difficult to qualitatively assess the effectiveness of a teaching method. The implementation of a new teaching method is associated with an inherent degree of bias, which makes the evaluation of such methods difficult. However, authors continue to seek for the appropriate method of anatomy teaching regardless of how best to achieve and validate it. Nevertheless, the best method to validate the effectiveness of such teaching approaches is to compare the students’ performance and results of assessments between schools employing different teaching methods. This may be difficult owing to the “multi-modal” approach that many medical schools use in their teaching and assessment modalities of anatomy [22]. 

Consequently, many choose to detect the attitude and perceptions of medical students towards cadaveric dissection and the newly implemented modalities for anatomy teaching. Two studies conducted by Patel and Moxham and Kerby and his associates have explored the perceptions of anatomists and medical students towards anatomy teaching methods. Both studies revealed that cadaveric dissection is preferred among anatomists and medical students in achieving the desired objectives predetermined among students. The results of both studies were not considered a clear distinction as the aims were based upon individual preferences of participants. They employed a “fitness for purpose” analysis method for implementing their results, which may not add a strong statistical significance of results [9, 15]. 

In the current work, we applied a more statistical approach to evaluate the differences in results between students who studied anatomy by two different methods (dissection or prosections with models) and attempted to detect the possible association between time spent in anatomy labs practicing dissection or studying prosections with models and the final course results of students. We found that students who learned anatomy by practicing dissection achieved better results (169 ± 1.99) in their final assessments than those who studied it by only using prosections and anatomical models (142 ± 1.78, p <0.001), The effect size of practicing dissection on final scores of students was high as d=2.08. This highlights that students may learn more from cadavers. In light of our findings, the concern that dissecting human cadavers is emotionally traumatic to students seems irrelevant [23].

In addition to this, practicing dissection increases cognitive abilities and attention span of medical students, besides increasing their physical endurance - an essential prerequisite for medical students [24]. This is similar to what Agius et al. stated that a strong interest in studying medicine as a career motivates students and lowers the level of their mental stress [25].

In our study, using prosections and anatomical models was not associated with high achievements in final assessments results, this is in accordance with previous studies that have reported students’ preference to practice dissection than using models and prosections as it enhances better understanding of course objectives and confers better three-dimensional appreciation of human anatomy [26]. Chapman et al. reported that applying new innovative approaches such as computer programs, models, and radiological anatomy techniques poorly scored compared to the traditional method of dissection. They also mentioned that these alternative approaches play an important role in complementing and reinforce the “core” teaching method [22].

However, previous studies have shown a very minimal difference in scores between students practicing dissection and those who learned anatomy by using prosections and anatomical models though learning from dissected cadavers is the satisfactory method of study. Recent studies show that students favor prosections and models than using cadavers, and some anatomists believe that prosections can replace full-body dissection in coming years especially in those institutions that have adopted an integrated curriculum and PBL as a teaching-learning method and this may be attributed to limited time for practicing dissection [27, 28].

A parallel association was found between time spent in practicing dissection and the final assessment results (r^2^ =0.841, p<0.001). However, in the prosections and models' group, there was no association between the time spent in studying prosections and final students' achievements (r^2^=0.002, p=0.735). The results of this study can be generalized to other institutions using different teaching methods. Still, to our knowledge, no previous study was performed to explore such results. Dusseau et al. in their study found that physical examination skills correlate with anatomy studies leading to a small improvement in anatomy scores [29, 30]. This is related to the learning of clinical skills and their role in improving the medical schools’ study curriculum.

The study has limitations: firstly, inherent errors in the measurement of assessment of anatomical knowledge may influence the results and efficacy of any such interventional study exercises. Secondly, medical students are inherently high performers due to their highly selective criteria for admission to a medical course; hence may not be affected by such interventional methods either way, as they may compensate anyway by their motivation to score higher in their courses. Thirdly, a baseline test (pre-exam), before starting the course (whether in dissection or prosection groups), was to be helpful to determine whether these two groups had a similar baseline level of anatomical knowledge prior to participating in the study. Furthermore, carrying out such a comparative study on one region only (upper limb) and conducting these optional courses during the summertime (hot climate) may have added some limitations to the study. Finally, despite our interesting findings, the sample size of the study groups is relatively small, and all cohort groups were only males. The gender mix in study groups was not possible as male and female students are completely separated for academic courses at all levels in our institution. Separate analysis for female students could be performed later, and the statistical significance of results could be considered for non-overlapping confidence intervals between study means. Further studies on a wider scale and on both males and females need to be conducted.

## Conclusions

In the current study, we aimed to evaluate the possible association between time spent in practicing dissection in anatomy classes and the performance of students in final assessments. In addition, we highlighted the significant difference in results between two groups who studied anatomy either by dissection or by using prosections and anatomical models. We tried keeping many variables nearly constant to add more validity to our results as students were assigned to each group randomly with nearly equal educational background. The assessment tools were the same for both groups. We assumed that only one variable, which was time spent in studying anatomy by dissection or by using models and prosecution, minimizes the interdependency of multiple variables and their relative contributions to students’ outcomes. More studyes are encouraged to measure not only the statistical significance of results but also their educational significance with reference to implementing significantly relevant changes in the strategies to be adopted in the teaching and learning of anatomy, both as basic science and its relevance to clinical practice. We believe that it would be challenging to analyze the effectiveness of different teaching methods, and hence anatomists are advised to exert more educational research.
